# Comprehensive Survey of Area-Wide Agricultural Pesticide Use in Southern United States Row Crops and Potential Impact on Honey Bee Colonies

**DOI:** 10.3390/insects10090280

**Published:** 2019-09-02

**Authors:** Jon Zawislak, John Adamczyk, Donald R. Johnson, Gus Lorenz, Joe Black, Quinton Hornsby, Scott D. Stewart, Neelendra Joshi

**Affiliations:** 1 Department of Entomology and Plant Pathology, University of Arkansas System, Division of Agriculture, Little Rock, AR 72204, USA; 2 Southern Horticultural Laboratory, Agricultural Research Service, United States Department of Agriculture, Poplarville, MS 39470, USA; 3 Department of Entomology and Plant Pathology, University of Arkansas System, Division of Agriculture, Lonoke, AR 72086, USA; 4 Department of Entomology and Plant Pathology, Department of Entomology and Plant Pathology, The University of Tennessee, Knoxville, TN 37996, USA; 5 Department of Entomology and Plant Pathology, University of Arkansas, Fayetteville, AR 72701, USA

**Keywords:** Apoideae, honey bee, *Apis mellifera*, pesticide, neonicotinoid, agriculture, pollinator decline, landscape, crops

## Abstract

Honey bees forage across a large area, continually scouting the local landscape for ephemeral food resources. Beekeepers often rely on flowering plants in and around irrigated farmland to maintain their colonies during dry seasons, despite the potential risk of pesticide exposure. Recent declines in pollinator abundance and diversity have focused attention on the role of pesticides and their effects on honey bee health. This investigation examined two types of landscapes within a two-mile (3.2 km) radius of honey bee colonies: an intensive agricultural setting and a rural setting without intensive agriculture. More than 10,000 acres of agricultural land was surveyed to quantify the area of cultivated crops and the area treated with pesticides, including seed treatments and foliar applications of insecticides. Samples of honey, bee bread (stored pollen), beeswax, and adult bees were collected from hives in both landscape types and screened for pesticide residues to determine if foraging bees were transporting pesticides to hives. Some samples of bee bread and honey did contain pesticide residues, but these were below known lethal dose (LD_50_) levels for honey bees. Beeswax samples contained the highest levels of contamination, but most were still relatively low. Samples were screened for 174 common agricultural pesticides and metabolites, but only 26 compounds were detected during the two-year study. These included one defoliant, one insect growth regulator, five herbicides, six fungicides, six insecticides never used in beekeeping, and five insecticides/miticides and their metabolites, which are used in beekeeping and for various other agricultural purposes, as well as two miticides exclusively used by beekeepers to control *Varroa destructor*. Bee colonies foraging in agricultural landscapes are potentially exposed to numerous pesticide applications. While the residues detected in this study did not pose an acute lethal risk to adult honey bees, this study did not measure sublethal effects on bee colony health or performance, which merit further investigation.

## 1. Introduction

Honey bees (*Apis meliffera* L.) are known to forage for food across an extensive landscape, up to three miles (5 km) or more from their hives [1]. While foraging distances are highly variable in different landscapes and in different seasons, as long as adequate resources are available, foragers tend to remain closer to their hives in order to conserve energy, within an average distance of about one mile (1.6 km) or less, and sometimes only a few hundred yards in agricultural settings with abundant food [2,3,4]. However, bees can range much farther for highly desirable food [5]. Honey bees exhibit preference for visiting flowers with high sugar content in the nectar, and will fly farther for higher quality forage, while bypassing lower quality forage nearby if the net caloric gain is greater [6]. Honey bees appear to be able to differentiate, and actively diversify their foraging, to compensate for protein deficiencies in dietary pollen [7,8]. Also, the floral resources available to bees are often ephemeral, with some species blooming for only a short time each season. For these reasons, bees continuously scout their territory to readily and efficiently exploit new sources of food before competitors [1].

The foraging activities of bee pollinators affect the continued survival of plant species as well as the genetic structure of distinct plant populations. Pollinator preferences have likely been a long-term driver of angiosperm speciation and evolution [9]. Both the long-range foraging habits of honey bees, and the relatively limited foraging range of solitary bee species, may be essential to the survival of plants in disturbed or fragmented habitats [10,11], such as those surrounding agricultural production areas. Small uncultivated areas within crop production landscapes can also serve as important refuge habitats for pollinators and other beneficial insects, as well as other wildlife species [12,13,14]. Many agricultural crops rely on insect pollination, either partially or completely, to ensure fruit and/or seed production [15]. Cereal grains such as corn, wheat, and rice are primarily wind-pollinated and do not require insect visits [16], although bees may sometimes collect their pollen for food [17]. Some large-scale commodity crops such as cotton and soybeans can be self-fertile and do not require insect pollination to produce yield, but there is some evidence that pollinator visits can increase yield production [18,19,20,21].

Commercial beekeepers often rely on irrigated farmland to sustain large numbers of honey bee colonies, and to produce surplus honey during dry periods, which would otherwise be a nectar dearth outside of an agricultural setting [22]. The amount of honey that these colonies can produce is affected by multiple factors that can determine nectar production, including cultivar variety, soil conditions, and weather [23,24]. While large-scale plantings of flowering crops can be significant nectar sources, bees in agricultural areas also greatly benefit from the presence of diverse wild flowers (i.e., weeds), which are also sustained on and around farms through dry conditions by crop irrigation. These plants can provide bees with additional pollen and nectar resources when crops may not be in bloom or when monocultures may not provide sufficient nutrition on their own [25,26]. Sponsler and Reed [27] reported that wax production and food accumulation were both positively correlated with proximity to crop land, as opposed to urban area, forest, or grassland. While intensive agricultural landscapes can greatly benefit honey bee colonies, beekeepers who maintain colonies in these areas must also be constantly wary of pesticides that can negatively affect their bees.

When foraging in an agricultural landscape, honey bees are potentially exposed to numerous insecticides, fungicides, herbicides, and other agricultural chemicals. Recent widespread declines in bee populations across the country have focused public scrutiny on the negative effects that agricultural chemicals may have on pollinator health [28,29]. Due to their widespread use in agriculture, especially as a pre-planting seed treatment, the neonicotinoid group has received particular attention because of suspected associations with declines in honey bee populations and health. These systemic insecticides can be translocated through the plant and into pollen and nectar, which becomes available to pollinating insects in sublethal quantities, which can negatively affect the behavior, reproduction, and survival of honey bees [30,31,32,33] and bumble bees [34,35].

The mid-South region of the United States has abundant agriculture as well as an abundance of diverse agricultural pests. Intensive crop production involves the diligent and routine scouting of fields for insects, weeds, and diseases, which are conventionally managed with a variety of insecticides, herbicides, and fungicides. Pesticide application decisions are routinely based on monitoring by crop consultants who determine appropriate pest control strategies. Honey bees from colonies in agricultural areas that are exposed to pesticides may transfer these compounds into the hive, potentially affecting the entire colony. When principles of integrated pest management (IPM) are followed, and pesticides are applied only on an as-needed basis, pests can be controlled while reducing off-target exposure to pollinators and other beneficial arthropods [36]. However, even with careful use, some level of exposure will likely be inevitable.

Pollen and/or bee bread collected from hives in numerous locations in France revealed contamination from multiple pesticides [37]. Bernal et al. [38] evaluated the pesticide residues in stored pollen from honey bee colonies in Spain, and found varying concentrations of numerous residues in both spring-collected and fall-collected samples. Mullin et al. [39] analyzed samples of beeswax, pollen, and honey bees from across North America, and detected 121 pesticides and their metabolites, with most samples containing multiple residues. In all of these studies, among the most prevalent residues detected were products routinely applied to hives by beekeepers for the control of Varroa mites, although some of these products have other pest-control applications as well. In Canada, Codling et al. [40] reported the detection of neonicotinoid insecticides and their breakdown metabolites in honey, pollen, and honey bees, although concentrations in most samples did not approach oral LD_50_ values for honey bees. That investigation did not screen for other classes of pesticides.

The current study describes the potential chemical exposure within the foraging territory of bee colonies located in an agricultural setting in the southern United States. The study sites were selected to represent the diversity of mid-South agriculture as well as areas with little or no agriculture. The crops in the intensive agriculture area were primarily soybeans, rice, corn, and cotton, with a few other minor crops, which included grain sorghum and green beans. Growers utilize a diverse selection of pesticide products for conventional production in Arkansas and the mid-South region, including herbicides, fungicides, and insecticides (including neonicotinoids as both as seed-treatments and foliar applications). A detailed survey was conducted to determine which crops were grown, and which pesticides were applied, across the entire landscape within a two-mile radius around an apiary. Sample of bees, beeswax, honey, and pollen were also collected from hives and screened for the presence of pesticide residues to which worker bees may have been exposed during foraging activity, and may have been brought back to the hive in collected food.

## 2. Methods and Materials

**Figure 1:**
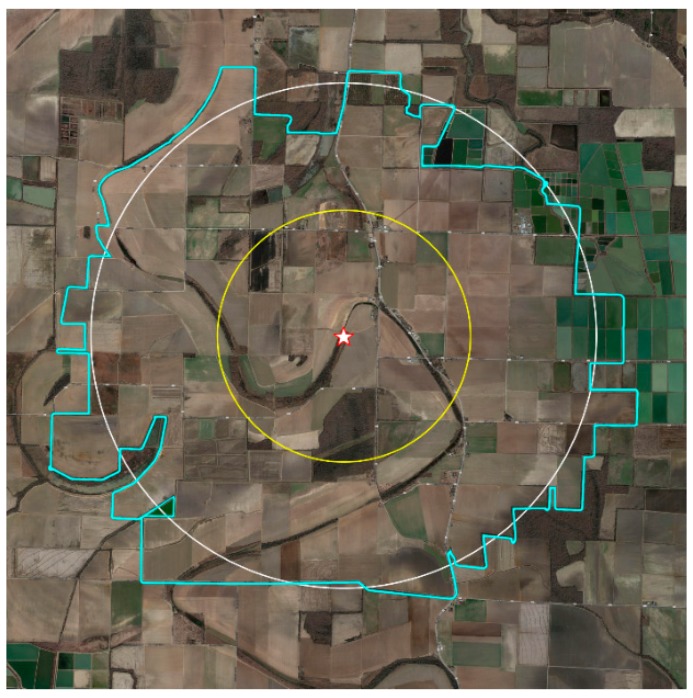
Aerial view of the High-Ag study site in Lonoke County, Arkansas. The star indicates the apiary location. The yellow circle indicates a one-mile radius from the beehives; the white circle indicates a two-mile radius from the hives; the blue line indicates the approximate area included in the survey. Landscape included the commercial production of soybeans, corn, rice, cotton, grain sorghum, and green beans, as well as commercial fish ponds, woodlands, grasslands, wetlands, and fallow fields. This site is representative of agricultural production land in this region (data: Google, Landsat/Copernicus, Maxar Technologies, US Geological Survey).

The survey was conducted in Lonoke County, Arkansas, during the 2014 and 2015 growing seasons. An apiary (“High-Ag” site) was established in April 2014, in an area where more than 80% of the landscape was under cultivation using conventional agricultural crop production methods and pesticide use. This site was representative of conditions around honey bee colonies in agricultural areas in the region. Four bee colonies were established in new 10-frame Langstroth beehives (two deep hive bodies each), using wired-beeswax foundation. All the beehive equipment was purchased from The Walter T. Kelley Company (Clarkson, KY, USA). Hives were protected from drift on all sides by a tree line, but bees had easy flight access to extensive cultivated row crop landscape in all directions (Figure 1).

**Figure 2:**
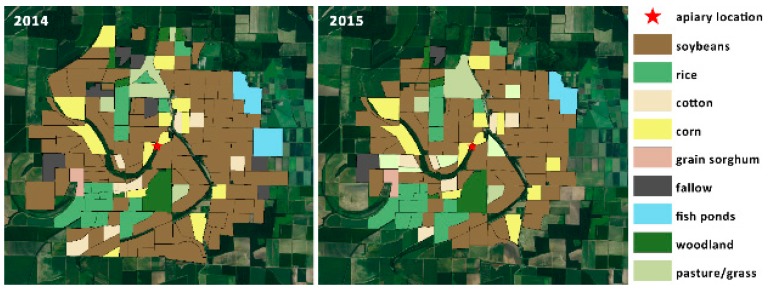
Land use by crop within the surveyed area around the High-Ag site during the 2014 and 2015 growing seasons. The survey area was slightly different between years due to changes in land use and an inability to contact farmers for interviews regarding all fields. However, general patterns of land use and crop production remained similar in the landscape around the apiary during both years.

A second apiary (“Low-Ag” site) was established at the same time, with four colonies, using identical equipment from the same sources. The Low-Ag site was also in Lonoke County, approximately 20 miles (32 km) from High-Ag site. The Low-Ag landscape was composed primarily of native grasses and forbs, pasture land, woodland, and some commercial fish farms, but was not surrounded by intensive row crop production (Figure 2).

The two sites were chosen for comparison because they were close together, with similar climate conditions, but surrounded by very different land use. Commercial beekeepers in the region favor apiary locations adjacent to agricultural land for higher honey production over non-agricultural land, despite the risk of pesticide exposure [22].

In 2014, all the colonies in both locations were started from three-pound packages purchased from the same source. In April 2015, eight additional colonies were established at the High-Ag site from locally-sourced nucleus colonies, and transferred into new, identical hives from the same source, as in 2014.

All the colonies, both years, were initially provided with 1:1 (sugar:water) syrup ad libitum for 1 month to help them establish and produce fresh comb. After this initial period, colonies foraged within the surrounding landscape for all their nutritional needs. All the colonies were managed with standard practices, for normal honey production, with additional hive bodies added as necessary. Queen excluders were not used, so that brood nest expansion was unlimited. No varroa control products were applied in 2014 prior to hive product sampling. Thymol (Apiguard^®^, Vita (Europe) Ltd., Basingstoke, UK) was applied, following label instructions, after hive products were sampled in 2014. In 2015, all the new nucleus colonies had been treated with amitraz (Apivar^®^, Véto-pharma, Palaiseau, France) for early season Varroa mite control prior to our purchase of them. Thymol (Apiguard^®^) was applied to all the colonies on 20 August, according to label instructions, approximately 5 weeks prior to taking hive product samples.

A map was created of the area surrounding the High-Ag apiary, and all the agricultural fields within a 2-mile (3.2 km) radius of the apiary were defined and measured using ArcGIS software (Esri, Redlands, CA, USA). If fields extended beyond this radius, the acreage of the entire field was included. While the actual honey bee foraging territory is potentially much larger than the acreage surveyed, land-use and farming practices are fairly consistent throughout the area surrounding the study site; therefore, the surveyed area is representative of the conditions that foraging honey bees would encounter in the local landscape outside of the survey radius.

Each crop field within the High-Ag study site was visually inspected to determine which crops were planted for two growing seasons. Growers were personally contacted and surveyed regarding their application of insecticides on each field. The survey determined only the presence of compounds (active ingredients) and/or specific product names that were applied. Information on the application rates, number or timing of applications made to all fields, and methods of application were not collected. The information gathered was limited to that which was voluntarily supplied by growers. While this data is likely incomplete, it does represent a minimum indication of the presence of these compounds applied to this landscape. The use of insecticide seeds treatments at planting was noted, and included as an application. Herbicide applications were not included in the survey, but were likely applied to most fields as a standard practice. Particularly, glyphosate (Roundup^®^, Bayer Ag, Leverkusen, Germany) was assumed to have been applied to most crops with engineered tolerance (soybean, corn and cotton), except for rice, green beans, and grain sorghum.

A map of the Low-Ag landscape was also made, and land use was calculated. An extensive survey of landowners in this area was not conducted, because this area did not contain significant large-scale row crop acreage. The majority of the landscape was pasture and woodland, but also contained a small fruit and pecan operation, some home gardens, a small dairy farm, and some commercial fish farming within the bees’ foraging range. While the fish farm could have been utilized as a water source by the bees, it is unlikely, as there were numerous fresh water sources (creeks and ponds) much closer to the apiary. Some soybean production was located approximately 2.5 miles (4 km) from the apiary, and an area of wheat was located approximately 1.5 miles (2.4 km) away, which was likely ignored by bees for lack of nectar. No other row-crop agriculture was located in the vicinity.

Samples were collected from bee hive products to determine if field-applied agricultural pesticides could be detected in beehives. Prior to colony installation in 2014, two samples of beeswax foundation were collected. Pieces of beeswax were sampled from 10 randomly selected sheets of wax foundation, which were part of a bulk purchase from which all the foundation used in the study originated. Additionally, two samples of adult bees were pooled from random packages at the time of colony installation. Later in the season, additional samples were taken from hives in both study apiaries (High-Ag and Low-Ag) in 2014. These samples included newly drawn beeswax comb (not yet used for brood-rearing or food storage, removed avoiding the foundation wax), bee bread (stored pollen), and adult honey bees randomly collected from inside the hive. Each sample consisted of 3–4 g of material or bees. All the samples were collected with sterile instruments, immediately placed on ice in the field, and later stored at −12 °C. Samples were shipped frozen, with dry ice, to the USDA’s National Science Laboratory in Gastonia, North Carolina, for their comprehensive apicultural pesticide screening. Sampling of live bees and hive products was repeated in 2015 only at the High-Ag site.

During 2014, samples for residue testing were collected on 6 August, and again on 24 September. On 6 August, samples of new beeswax, bee bread, and adult bees were collected from each of two hives at the High-Ag site and from each of two hives at the Low-Ag site. On 24 September, the sampling procedure was repeated from each of the same hives at both sites, with capped honey also collected from each of the same hives.

In 2015, samples of adult honey bees and beeswax from combs in nucleus colonies were collected when the colonies were initially established. However, these samples were accidently destroyed in shipment, and could not be analyzed for residue contaminants. Additional samples of hive products were collected on 29 September from 4 hives in the High-Ag area. The samples included new beeswax, bee bread, honey, and adult bees. Colonies in the Low-Ag area were not sampled in 2015, because none of the Low-Ag samples from 2014 contained detectable residues except for the new beeswax, which contained only very low levels. Resources were instead devoted to samples taken in the High-Ag apiary.

## 3. Results and Discussion

**Figure 3:**
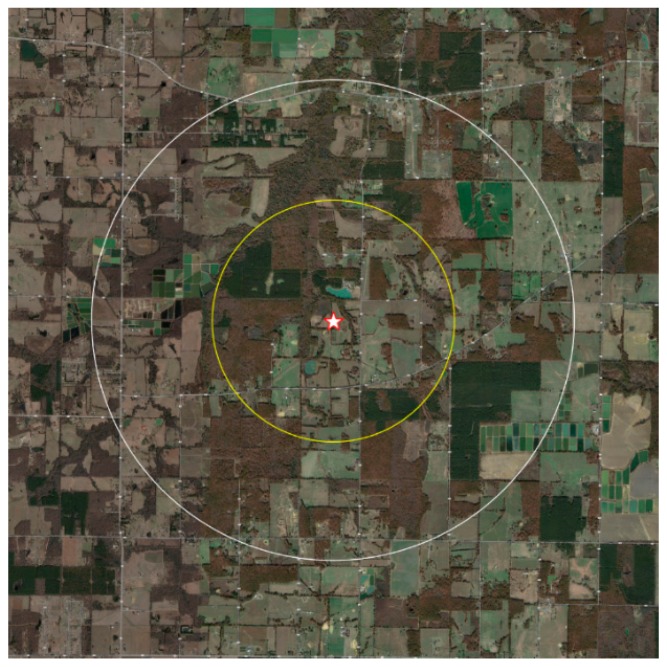
Aerial view of the Low-Ag study site, Lonoke County, Arkansas. The star indicates the apiary location. The yellow circle indicates a one-mile radius from the beehives; the white circle indicates a two-mile radius from the hives. The landscape included a diverse mixture of pasture, woodlands, commercial fish farming, residential gardens, and a few small fruit or orchard operations, but no significant row crop agriculture near the apiary site (data: Google, Maxar Technologies, State of Arkansas, USDA Farm Services Agency).

**Figure 4:**
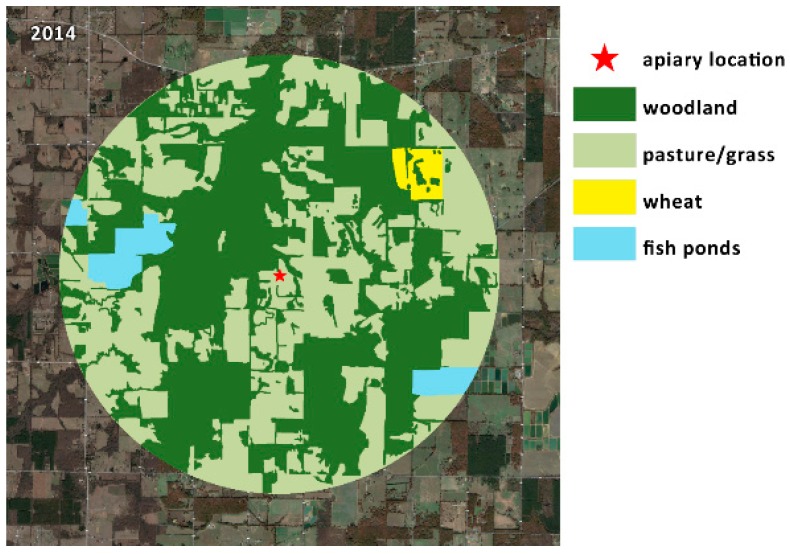
Dominant land use within a two-mile radius around the Low-Ag site in 2014. This landscape was primarily composed of woodland and grassland/pasture, with a small area of wheat, and some commercial fish farming.

The survey of the High-Ag landscape included all the area within a two-mile radius of the apiary (8038 acres). If cultivated fields extended beyond this radius, the entire field was included. The total surveyed area under cultivation varied between 2014 (12,160 acres) and 2015 (10,063 acres). The total area of the survey was slightly different between years because of changes in land management, and an inability to contact some growers for interviews. The aerial map in Figure 1 shows the High-Ag area surveyed, in the context of its surrounding landscape. Crops in the High-Ag area included a predominant commercial production of soybeans, corn, rice, cotton, and grain sorghum, as well as small areas of green beans, some commercial fish farming, woodland, wetlands, pasture, and fallow fields, which are typical of this area. The maps in Figure 2 indicate the distribution of land use by crop around the High-Ag site for both years. Slight changes in land use between growing seasons did occur, but did not significantly modify the overall composition of the landscape. Figure 3 shows an aerial view of the landscape around the Low-Ag apiary site, which was dominated by a mixture of pasture and woodlands, with some small home gardens, commercial fish farming, and a few small fruit operations, but very little row crop agriculture. Figure 4 outlines the dominant land use within a two-mile radius of the beehives.

An average of 81% of the landscape was under cultivation in the High-Ag area during the 2014 and 2015 growing seasons (Table 1). The largest proportion (57%) was planted with soybeans, while 10% was used for rice, 8% was used for corn, and 6% was planted with minority crops (cotton, grain sorghum, green beans). The remaining landscape was comprised of 15% uncultivated land (fallow fields, pasture, woodland, wetland), with 4% devoted to commercial fish ponds. This extensive agricultural area supplied bee colonies with ample forage to build up population numbers and produce surplus honey, but also had potential for significant exposure to numerous pesticides applied throughout the season. Grower-reported applications of insecticides and fungicides in 2014 and 2015 are summarized by crop in Table 2.

The Low-Ag site, within two miles (3.2 km) of the apiary, had very little of the landscape devoted to row crop agriculture (Table 3). Less than 6% of the landscape was devoted to wheat—which is not attractive to honey bees—and fish farming. The rest of the land around the site was either woodland (54%) or grass/pasture (43%). Pastures may contain bee-attractive flowers, and are sometimes treated for fall armyworms to protect grazing and hay crops, but no products recommended for armyworm control [41] were detected in any of our samples.

**Figure 5:**
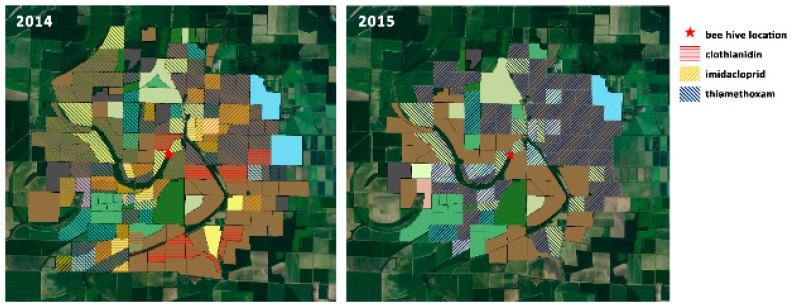
Reported distribution of neonicotinoid insecticides applied as seed treatments within the High-Ag survey area during the 2014 and 2015 growing seasons.

**Figure 6:**
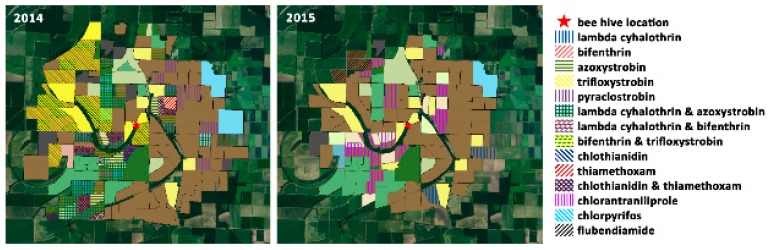
Reported distribution of foliar applied pesticides in the surveyed area within the High-Ag survey area during the 2014 and 2015 growing seasons.

Figure 5 illustrates the reported distribution of crops planted with neonicotinoid seed treatments. These treatments have come under particular scrutiny for their potential to translocate toxins and make them available to foraging bees in pollen and nectar, however Stewart et al. reported generally low concentrations of these products when sampling seed-treated crops growing in the mid-South [42]. Figure 6 illustrates the distribution of foliar pesticide applications reported around the apiary site.

Samples of package bees and beeswax foundation were taken when colonies were established and screened for pesticide residues along with hive products sampled later in the season. Both the package bees and foundation wax contained compounds that we had not applied to the hives, and were not reported as used by area farmers, but were detected (Table 4). Coumaphos and fluvalinate were both detected in package bees, which could be a result of the package bee supplier treating bees for mites prior to shipping spring packages. The presence of the herbicide atrazine in package bees is curious, and may have resulted from bees encountering the compound prior to being packaged for sale.

The highest levels of residues found in wax foundation were coumaphos and fluvalinate, which agrees with Mullin et al. [39] and Medici et al. [33]. These products are commonly applied by beekeepers to control Varroa mites. These lipophilic compounds are known to be readily soluble in beeswax [43,44], and remain stable when wax is melted and formed into new foundation sheets [45]. Chlorpyrifos was also detected, but at a much lower level than that found by Mullin et al. [31].

Samples of adult bees and drawn comb were also initially collected from nucleus colonies established in the High-Ag apiary in 2015; however, these samples were accidently destroyed in shipping, and could not be analyzed for residues.

Given that agricultural pesticides were routinely applied to much of the landscape around the apiary, we expected that bees would be exposed to these while foraging, and had potential to transport contaminated nectar or pollen back to the hive. Samples of beeswax, bee bread, honey, and bees were screened for 174 common agricultural pesticides and their metabolites. Of these, only 26 compounds were detected during the two-year study, including one defoliant, one insect growth regulator, five herbicides, six fungicides, six insecticides never used in beekeeping, and five insecticides/miticides and their metabolites which are used in beekeeping and for various other agricultural purposes, as well as two miticides exclusively used by beekeepers to control *Varroa destructor*. Overall, considering the widespread use of pesticides in the landscape around the apiary at the High-Ag site, bee hive samples contained fairly little contamination. The residues detected in hive samples are summarized in Table 5. A list of the compounds screened, but not detected, is reported in Table 6.

In honey sampled at the High-Ag site, the only contaminants detected were flubendiamide (in 2014) and DMPF (2,4-dimethylphenyl formamide) (in 2015). This agrees with Rissato et al. [46] and Alburaki et al. [47], who also found pesticide concentrations in honey to be very low or undetectable. This is likely because many synthetic pesticides are lipophilic, and readily accumulate in beeswax [44], but are not especially soluble in honey [45]. Also, many foliar-applied insecticides work by contact, and are unlikely to be present in nectar collected by bees. Honey samples from the Low-Ag site contained no detectable residues.

Bee bread collected from hives in the High-Ag apiary contained four compounds in 2014 and three compounds in 2015, but all at low levels. A review by Bogdanov [48] also suggests that pollen (bee bread) is more likely to be contaminated with residues than honey. Bee bread samples from the Low-Ag site contained no detectable residues.

No pesticide residues were detected in adult bee samples in 2014, from either the High-Ag or Low-Ag sites. However, because adult bees are short-lived in the summer, our limited sampling at the end of the season may not have detected applications made earlier. Similarly, in 2015, only beekeeper-applied products were detected in adult bee samples.

New beeswax contained the highest number of detected compounds at both sites, and in both years. New beeswax sampled from the Low-Ag site in 2014 contained the highest number of compounds detected (16). The sources of these contaminants in the Low-Ag landscape are unknown, but were generally well below LD_50_ values for bees. In new beeswax sampled at the High-Ag site, nine compounds were detected in 2014, and seven compounds were detected in 2015.

In 2015, a high level of the herbicide metolachlor was detected in samples of new beeswax, but not in bee bread or honey. This contamination could have been the result of foraging honey bees in contact with freshly applied material, and spreading it to wax while walking across the comb. Several fungicides were detected, again mostly in beeswax. These are commonly used to control blight and plant diseases in agriculture, and are not presumed to be acutely toxic to honey bees. However, when synergized with other compounds, the combined toxicity may increase [39,49,50]. Also, exposure to fungicides appears to make honey bees more susceptible to the gut pathogen *Nosema cerana* [51]. Also, acute toxicity is not the only concern of pollinator health. Numerous sublethal effects from exposure to single and multiple pesticides have been noted in recent literature [28,33,52,53,54,55].

The highest levels of residues detected in wax were from products that are primarily applied by beekeepers for Varroa mite control. In 2014, coumaphos and fluvalinate were detected in new beeswax at both sites. Both of these compounds had been detected in foundation wax and package bees at the beginning of the season, but were not applied early to hives during the experiment, and were not likely to be used for any nearby field application. Both of these are known to migrate from contaminated wax [52]. Their presence in newly secreted beeswax suggests that these lipophilic chemicals may have diffused from contaminated foundation or been spread by contact with the bodies of bees. In 2015, wax samples contained residues of products that were applied to colonies. Amitraz had been applied for Varroa control in nucleus colonies prior to purchase, according to the nucleus colony provider. No amitraz was detected in the subsequent sampling of any hive products, but DMA (2,4-dimethyl aniline) and DMPF, which are both breakdown products of amitraz [56], were detected more than six months later in samples of adult bees, capped honey, bee bread, and new beeswax. Also, high levels of thymol were present in adult bees that were sampled after Varroa control application of thymol was made in the late summer. However, thymol was not detected in other hive products. Thymol is a naturally derived essential oil that is obtained from the thyme plant (*Thymus vulgaris*), and not considered toxic to bees [57], but can affect the flavor of honey if applied before honey is harvested [58].

Absent from the list of detected compounds are any of the neonicotinoid group of insecticides, which have recently received much critical attention for their suspected role in honey bee population declines. Krupke et al. [59] suggested that dust exhausted during planting treated seeds could potentially contaminate nearby wildflowers where bees forage, which was confirmed by Stewart et al. [42]. Dively and Kamel [60] found that neonicotinoid treatments applied as foliar applications or through chemigation resulted in the highest residues in nectar and pollen in cucurbits, while the lowest residues were detected from seed dressings. Furthermore, Meikel et al. [61] found that imidacloprid remained stable in hive products for at least seven months. A worldwide survey of honey as a human food product found very low levels of neonicotinoid contamination, with a mean for positive detections of 1.8 ± 0.56 (SE) ppb [62]. In the current survey, neonicotinoid products were applied as pre-plant seed coatings (i.e., seed treatments) as well as via foliar applications on multiple crops throughout the foraging landscape around the High-Ag apiary site. Despite their widespread use in this landscape, we did not detect any neonicotinoids in our samples. However, our sampling was limited to the end of the growing season, when residues from early season treatments or other sporadic applications may not have been detectable.

## 4. Conclusions

Honey bees forage over an extensive area for the nectar and pollen they utilize as food. In agricultural landscapes, there is great potential for pesticide exposure of honey bees in the field, and for contamination of the hive and hive products. The Arkansas survey of area growers, although most certainly incomplete in documenting all pesticide applications, confirms that multiple products, in multiple chemical classes, are applied to the agricultural landscape routinely throughout the season as part of conventional agricultural production.

Despite the widespread use of these chemicals, both hobbyist and commercial beekeepers continue to maintain productive honey bee colonies in intensive agricultural areas [22]. Furthermore, colony productivity has been shown to increase with proximity to crop land [27], and research has also shown that mass flowering crops can benefit wild and managed bees, despite other risks posed by agricultural practices and land management [63,64,65].

The results of our limited investigation are consistent with other studies. Similar to Mullin et al. [31], who conducted one of the broadest and most geographically diverse studies, we found that the highest concentrations of detectable compounds were a result of beekeeper-applied products. These products, by design, have low toxicity relative to the dose required for adverse effects. To a lesser degree, fungicides and herbicides also have low general toxicity to honey bees, but are known to have synergistic effects with other pesticides, which increase the toxicity of one or more of the compounds [50,66,67]. The increasing buildup of pesticide contamination in combs over time can adversely affect honey bee health and survivorship [68,69,70]. Chronic exposure to sublethal levels of pesticides can impact honey bee health and immune response [51,71]. Pesticides are rarely, if ever, encountered individually, but more often simultaneously with others [39]. Efforts have been made to explore the toxicity of combinations of pesticides that are often found together [49,50,70,71].

Recent declines in honey bee populations cannot be attributed to any one single cause, but are likely the result of accumulated stresses from multiple causes [53]. The complex of the mite *Varroa destructor* (Anderson and Trueman) and the viruses they vector continues to be the greatest threat to honey bee health [72,73]. Other pathogens such as *Nosema ceranae* also affect honey bee health, productivity, and survivorship [74]. Additionally, bees must have access to adequate nutrition from floral resources in order to maintain health [75]. Most likely, a combination of multiple factors, including these and others, are responsible for recent declines in honey bee health and populations [53,76]. Optimal management of honey bee colonies must include a reduction of multiple stress factors, including sublethal exposure to pesticides, and discussions of honey bee health should not be limited to a narrow focus on pesticide exposure.

To expand upon this work, a similar survey could be conducted that includes records on the timing, formulations, and rates of pesticide applications for specific crop fields, and more frequent sampling through the season to more precisely determine when contaminants may be entering beehives, and how long particular applications may pose specific risks to bee colonies.

## Figures and Tables

**Table 1:** Summary of land use within the High-Ag survey site in 2014–2015. This site included all the agricultural fields within approximately two miles of the apiary location. Areas of crop fields that extended outside of a two-mile radius were included in the survey.

Land Use	Total Acreage		% Acreage		
	2014	2015	2014	2015	2-Year Average
Soybean	7489	5285	61.6	52.5	57.1
Rice	1110	1088	9.1	10.8	10
Corn	1005	849	8.3	8.4	8.4
Cotton	443	317	3.6	3.2	3.4
Grain Sorghum	92	91	0.8	0.9	0.9
Green Beans	0	306	0	3	1.5
Total Crop Acreage	10,139	7936	83.4	78.9	81.2
Fish Ponds	396	396	3.9	3.9	3.9
Uncultivated Land	1625	1731	12.7	17.2	15
Total Acreage	12,160	10,063	100	100	100

**Table 2:** Reported acreage receiving pesticide application, by crop, within the High-Ag survey area during the 2014 and 2015 growing seasons.

Year	Pesticide	Class *	Number of Acres of Each Crop Treated by Pesticide Listed						Total Acres Treated	Percentage Surveyed Landscape Treated
			Soybean	Corn	Rice	Grain Sorghum	Cotton	Green Bean		
2014	Thiamethoxam	i-neo	3677	789	669	92	264	0	5491	45.2
	Imidacloprid	i-neo	884	81	0	0	203	0	1168	9.6
	Clothianidin	i-neo	1054	81	0	0	11	0	1146	9.4
	Dimethoate	I-op	54	0	0	0	0	0	54	0.4
	Cypermethrin	I-py	33	0	0	0	61	0	94	0.8
	Lambda-Cyhalothrin	i-pyr	685	0	347	0	192	0	1224	10.1
	Bifenthrin	i-pyr	319	81	0	0	11	0	411	3.4
	Chlorantraniliprole	i-ry	319	50	0	0	72	0	441	3.6
	Flonicamid	i-u	175	0	0	0	10	0	185	1.5
	Novaluron	igr	285	81	0	0	11	0	377	3.1
	Fludioxonil	f	3637	868	669	92	192	0	5458	44.9
	Mefenoxam	f	3637	868	669	92	192	0	5458	44.9
	Azoxystrobin	f	1608	0	347	0	323	0	2278	18.7
	Prothioconizole	f	1567	509	62	0	0	0	2138	17.6
	Trifloxystrobin	f	1567	509	62	0	0	0	2138	17.6
	Metalaxyl	f	564	0	0	0	131	0	695	5.7
	Tebuconazole	f	564	0	0	0	131	0	695	5.7
	Tiabendazole	f	519	0	0	0	0	0	519	4.3
	Pyraclostrobin	f	479	0	0	0	0	0	479	3.9
	Propiconazole	f	0	0	292	0	0	0	292	2.4
2015	Thiamethoxam	i - neo	2965	0	344	0	317	225	3851	38.3
	Clothianidin	i - neo	0	849	0	0	317	0	1166	11.6
	Acephate	i - op	0	0	0	0	317	0	317	3.2
	Chlorpyrifos	i - op	0	0	0	91	0	0	91	0.9
	Bifenthrin	i - pyr	0	0	0	0	317	0	317	3.2
	Lambda-Cyhalothrin	i - pyr	199	0	0	0	0	0	199	2
	Chlorantraniliprole	i - ry	768	0	0	0	317	93	1178	11.7
	Flubendiamide	i - ry	256	0	0	0	0	0	256	2.5
	Novaluron	igr	0	0	0	0	317	0	317	3.2
	Fludioxonil	f	2197	0	0	0	0	132	2329	23.1
	Mefenoxam	f	2197	0	0	0	0	132	2329	23.1
	Azoxystrobin	f	877	312	745	0	0	306	2240	22.3
	Propiconazole	f	0	312	344	0	0	0	656	6.5

* f = fungicide, i = insecticide, igr = insect growth regulator; neo = neonicotinoid; op = organophosphate, pyr = pyrethroid, ry = ryanoid, u = unclassified.

**Table 3:** Summary of land use within a two-mile radius around the Low-Ag site in 2014.

Land Use	Total Acreage	% Acreage
Woodland	7489	54.0
Grass/Pasture	1110	42.5
Fish Ponds	1005	3.5
Wheat	443	1.2
Total Acreage	8043	100

**Table 4:** Compounds detected in initial samples of package bees and foundation wax used to establish colonies in 2014. Results reported as ppb, and are a mean of two separate samples randomly taken on the day of installation.

Compound	Class *	Level of Detection (ppb)	Beeswax Foundation	Package Bees
coumaphos	a	5	323.5	59
fluvalinate	a	1	273	136.5
chlorpyriphos	i	1	2.6	0
hexythiazox	igr	30	trace	0
vinclozolin	f	1	trace	0
atrazine	h	6	0	96.9

* a = acaricide, f = fungicide, h = herbicide, i = insecticide; 0 = not detected; trace = detected, but insufficient to quantify.

**Table 5:** Pesticide residues detected in hive products. Results are given in parts per billion (ppb, ±SE). Where results are reported as 0, compound was not detected. Where results are reported as “trace” the compound was detected, but at a level too low to be quantifiable.

Pesticide	Class *	Level of Detection (ppb)	2014				2015			
			Low-Ag	High-Ag			High-Ag			
			New Wax	Honey	Pollen	New Wax	Honey	Pollen	New Wax	Bees
Coumaphos	a	5	158.85 (95.38)	0	0	103.75 (73.08)	0	0	0	0
Coumaphos Oxon ***	a	5	1.28 (2.55)	0	0	trace	0	0	0	0
Fluvalinate	a	1	128.53 (61.1)	0	0	63 (73.52)	0	0	0	0
Amitraz	a	4	0	0	0	0	0	0	0	0
DMA **	a	50	0	0	0	0	0	0	0	297.5 (595)
DMPF **	a	10	0	0	0	0	13.05 (15.66)	0.38 (0.25)	769.75 (373.05)	trace
Thymol	a	50	trace	0	0	0	0	0	0	747.5 (1495)
Bifenthrin	i	2	37 (30.2)	0	4.98 (9.95)	3.75 (4.37)	0	2.05 (4.1)	14.3 (3.03)	0
Chlorpyrifos	i	1	0.68 (1.35)	0	0	0.55 (1.1)	0	0	0	0
Cyhalothrin	i	1	0.55 (1.1)	0	3.78 (0.79)	0	0	2.48 (2.94)	0	0
Dimethoate	i	50	0.25 (0.5)	0	0	0	0	0	0	0
Flubendiamide	i	25	0	48.7 (68.87)	0	0	0	0	0	0
Methyl Parathion	i	2	0.25 (0.5)	0	0	0	0	0	0	0
Hexythiazox	igr	30	0.25 (0.5)	0	0	0.5 (0.58)	0	0	0	0
Azoxystrobin	f	2	1.13 (2.25)	0	30.25 (36.07)	2.13 (4.25)	0	0	0	0
Carbendazim	f	5	0	0	0	0	0	0	0.25 (0.29)	0
Chlorothalonil	f	30	0	0	0	0.5 (0.58)	0	0	0	0
Metalaxyl	f	2	1.55 (3.1)	0	0	0	0	0	0	0
Trifloxystrobin	f	1	0.5 (0.58)	0	0	0	0	0	0	0
Vinclozolin	f	1	0	0	0	0.25 (0.5)	0	0	0	0
Atrazine	h	6	2.35 (4.7)	0	0	0	0	0	0.25 (0.29)	0
Metolachlor	h	6	0	0	0	0	0	0	241.25 (311.42)	0
Metribuzin	h	1	0	0	0	0	0	0	10.9 (5.01)	0
Pendimethalin	h	6	8.8 (16.94)	0	0	0	0	0	0	0
Tribufos	d	2	0	0	3.9 (7.8)	0	0	0	8.48 (16.95)	0

* a = acaricide, d = defoliant, f = fungicide, h = herbicide, i = insecticide, igr = insect growth regulator; ** DMA = 2,4-dimethylanaline, DMPF = 2,4-dimethylphenyl formamide; both are breakdown metabolites of amitraz; *** coumaphos oxon is a breakdown metabolites of coumaphos.

**Table 6:** All beehive samples were screened for 174 common agricultural chemicals and metabolites. Of these, 148 compounds that were not detected in any samples are listed, with their levels of detection (LOD) in ppb.

Compound	LOD	Compound	LOD	Compound	LOD
1-Naphthol	10	Dinotefuran	2	Parathion methyl	2
3-Hydroxycarbofuran	10	Diphenamid	20	Permethrin total	10
4,4 dibromobenzophenone	4	Endosulfan I	2	Phenothrin	10
4-Hydroxychlorothalonil	50	Endosulfan II	2	Phorate	50
Acephate	50	Endosulfan sulfate	2	Phosalone	10
Acetamiprid	2	Endrin	10	Phosmet	10
Acetochlor	50	Epoxiconazole	1	Piperonyl butoxide	50
Alachlor	10	Esfenvalerate	2	Pirimiphos methyl	20
Aldicarb	4	Ethion	10	Prallethrin	4
Aldicarb sulfone	2	Ethofumesate	10	Profenofos	10
Aldicarb sulfoxide	20	Etoxazole	1	Pronamide	1
Aldrin	10	Etridiazole	50	Propachlor	10
Allethrin	10	Famoxadone	20	Propanil	10
Amicarbazone	30	Fenamidone	10	Propargite	10
Azinphos methyl	6	Fenbuconazole	10	Propazine	20
Bendiocarb	10	Fenhexamid	6	Propetamphos	4
Benoxacor	20	Fenoxaprop-ethyl	20	Propham	20
BHC alpha	4	Fenpropathrin	10	Propiconazole	20
Bifenazate	20	Fenpyroximate	5	Pymetrozine	20
Boscalid	4	Fenthion	10	Pyraclostrobin	15
Bromuconazole	20	Fipronil	10	Pyrethrins	50
Buprofezin	20	Flonicamid	8	Pyridaben	10
Captan	10	Fludioxonil	20	Pyrimethanil	20
Carbaryl	30	Fluoxastrobin	4	Pyriproxyfen	10
Carbofuran	10	Fluridone	10	Quinoxyfen	10
Carboxin	4	Flutolanil	4	Quintozene (PCNB)	1
Carfentrazone ethyl	1	Heptachlor epoxide	10	Resmethrin total	5
Chlorfenopyr	1	Heptachlor	4	Sethoxydim	2
Chlorfenvinphos	6	Hexachlorobenzene (HCB)	1	Simazine	50
Chlorferone	50	Hydroprene	20	Spinosad	50
Chlorpropham (CIPC)	40	Imazalil	20	Spirodiclofen	2
Clofentezine	100	Imidacloprid 5-hydroxy	25	Spiromesifen	10
Clothianidin	1	Imidacloprid	1	Tebuconazole	8
Cyfluthrin	4	Imidacloprid olefin	10	Tebufenozide	10
Cypermethrin	4	Indoxacarb	3	Tebuthiuron	2
Cyphenothrin	20	Iprodione	50	Tefluthrin	1
Cyprodinil	1	Lindane	4	Tetrachlorvinphos	4
DDD p,p’	4	Linuron	20	Tetraconazole	6
DDE p,p’	2	Malathion	4	Tetradifon	1
DDT p,p’	4	Methamidophos	4	Tetramethrin	10
Deltamethrin	50	Methidathion	10	Thiabendazole	1
Diazinon	5	Methomyl	10	Thiacloprid	1
Dichlorvos (DDVP)	50	Methoxyfenozide	10	Thiamethoxam	1
Dicloran	1	MGK-264	50	THPI	50
Dicofol	1	MGK-326	10	Triadimefon	2
Dieldrin	10	Myclobutanil	15	Triadimenol	45
Difenoconazole	10	Norflurazon	6	Triflumizole	50
Diflubenzuron	10	Oxamyl	5	Triticonazole	10
Dimethenamid	10	Oxyfluorfen	1		
Dimethomorph	20	Paradichlorobenzene	10		
